# Supplementary material for: Oxidative Stress Markers in Human Brain and Placenta May Reveal the Timing of Hypoxic-Ischemic Injury: Evidence from an Immunohistochemical Study
Source: Int J Mol Sci. 2023 Jul 30;24(15):12221. doi: 10.3390/ijms241512221 (PMC10418753; doi:10.3390/ijms241512221)
Supplement: Supplementary file 1 [file ijms-24-12221-s001.zip › ijms-2439847-supplementary.pdf]

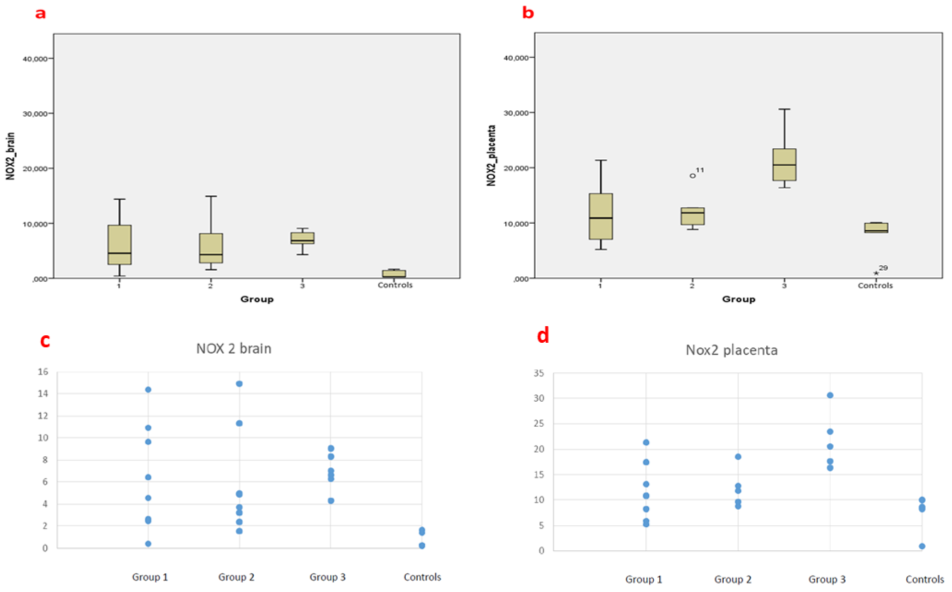

Figure S1: Box plots **(a-b)** and Dot plots **(c-d)** of the immunohistochemical expression of NADPH oxidase 2 (NOX2) among groups: Brain cortex (a-c); Placental tissue (b-d). Data reported as median with interquartile range (IQR) of the percentage of the number of positive colored cells/microscopic area analyzed. P values are calculated by Kruskal-Wallis test. Comparisons between two independent groups were made by Mann-Whitney U test. The graphical representation shows in the three cases Groups an evident positive reaction than the Control Group.

S1a. Kruskal-Wallis test: $p<0.05$; Mann-Whitney U test: Group 1, Group 2, Group 3 vs controls: $p<0.05$.

S1b. Kruskal-Wallis test: $p<0.05$; Mann-Whitney U test: Group 3 vs controls: $p<0.05$; Group 1 vs Group 3: $p<0.05$; Group 2 vs Group 3: $p<0.05$.

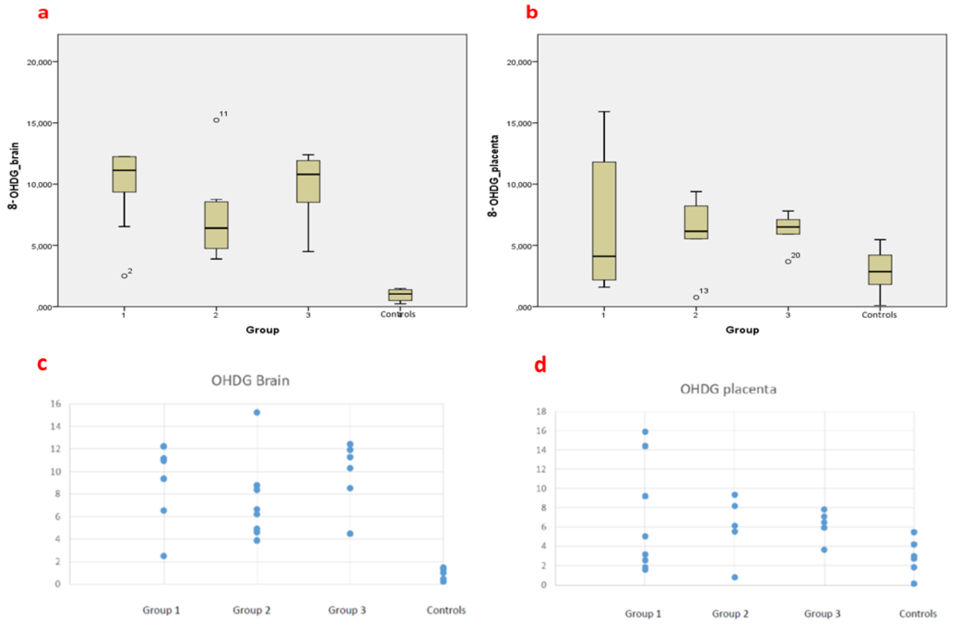

Figure S2.: Box plots **(a-b)** and Dot plots **(c-d)** of the immunohistochemical expression of 8-Hydroxy-2′-deoxyGuanosine (8OHdG) among groups: Brain cortex (a-c); Placental tissue (b-d). Data reported as median with interquartile range (IQR) of the percentage of the number of positive colored cells/microscopic area

analyzed. P values are calculated by Kruskal-Wallis test. Comparisons between two independent groups were made by Mann-Whitney U test. The graphical representation shows in the three cases Groups an evident positive reaction than the Control Group.

S2a. Kruskal-Wallis test: $p>0.05$; Mann-Whitney U test: Group 1, Group 2, Group 3 vs controls: $p<0.05$.

S2b. Kruskal-Wallis test: $p<0.05$; Mann-Whitney U test: Group 3 vs controls: $p<0.05$.

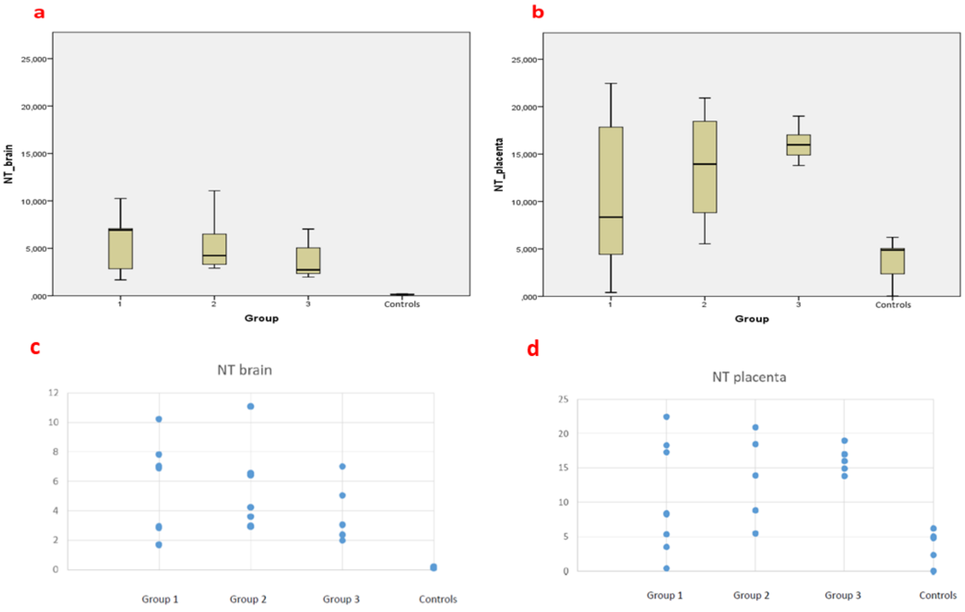

Figure S3: Box plots **(a-b)** and Dot plots **(c-d)** of the immunohistochemical expression of Nitro Tyrosine (NT) among groups: Brain cortex (a-c); Placental tissue (b-d). Data reported as median with interquartile range (IQR) of the percentage of the number of positive colored cells/microscopic area analyzed. P values are calculated by Kruskal-Wallis test. Comparisons between two independent groups were made by Mann-Whitney U test. The graphical representation shows in the three cases Groups an evident positive reaction than the Control Group.

S3a. Kruskal-Wallis test: $p<0.05$; Mann-Whitney U test: Group 1, Group 2, Group 3 vs controls: $p<0.05$.

S3b. Kruskal-Wallis test: $p<0.05$; Mann-Whitney U test: Group 2, Group 3 vs controls: $p<0.05$.

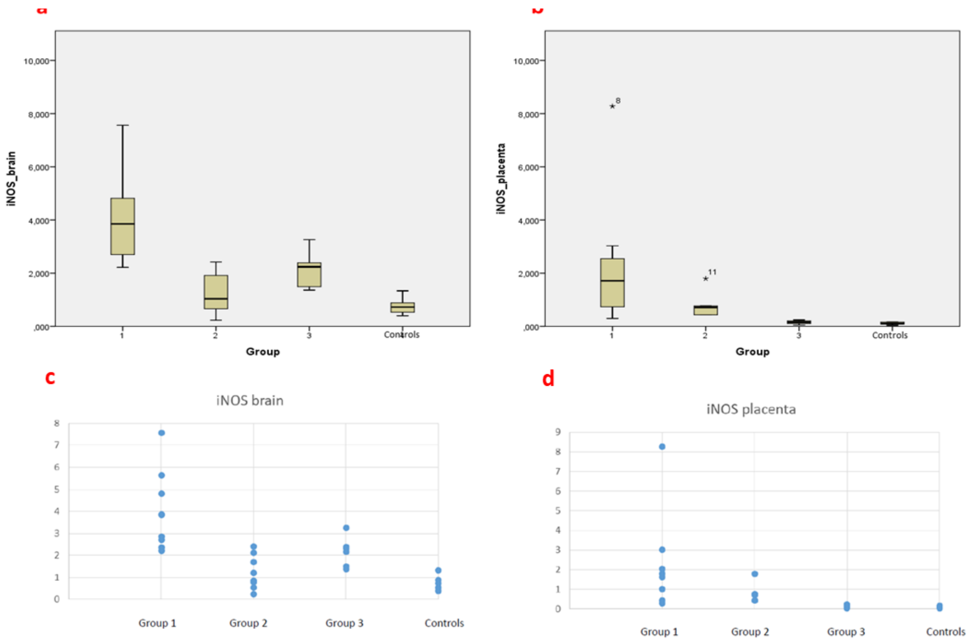

Figure S4: Box plots **(a-b)** and Dot plots **(c-d)** of the immunohistochemical expression of inducible Nitric Oxide Synthase (iNOS) among groups: Brain cortex (a-c); Placental tissue (b-d) Data reported as median with interquartile range (IQR) of the percentage of the number of positive colored cells/microscopic area analyzed. P values are calculated by Kruskal-Wallis test. Comparisons between two independent groups were made by Mann-Whitney U test. iNOS shows the highest expression in brain tissue Group_1 B followed by Group_3 B, the iNOS immunoreactivity was very low in Group_2 B and Controls; placental tissues of Group_1 P and Group_2 P showed higher expression of iNOS than Group_3 P and Controls.

S4a. Kruskal-Wallis test: $p<0.05$; Mann-Whitney U test: Group 1, Group 3 vs controls: $p<0.05$. Group 1 vs Group 2: $p<0.05$. Group 1 vs Group 3: $p<0.05$; Group 2 vs Group 3: $p<0.05$.

S4b. Kruskal-Wallis test: $p<0.05$; Mann-Whitney U test: Group 1, Group 2 vs controls: $p<0.05$. Group 1 vs Group 3: $p<0.05$; Group 2 vs Group 3: $p<0.05$.

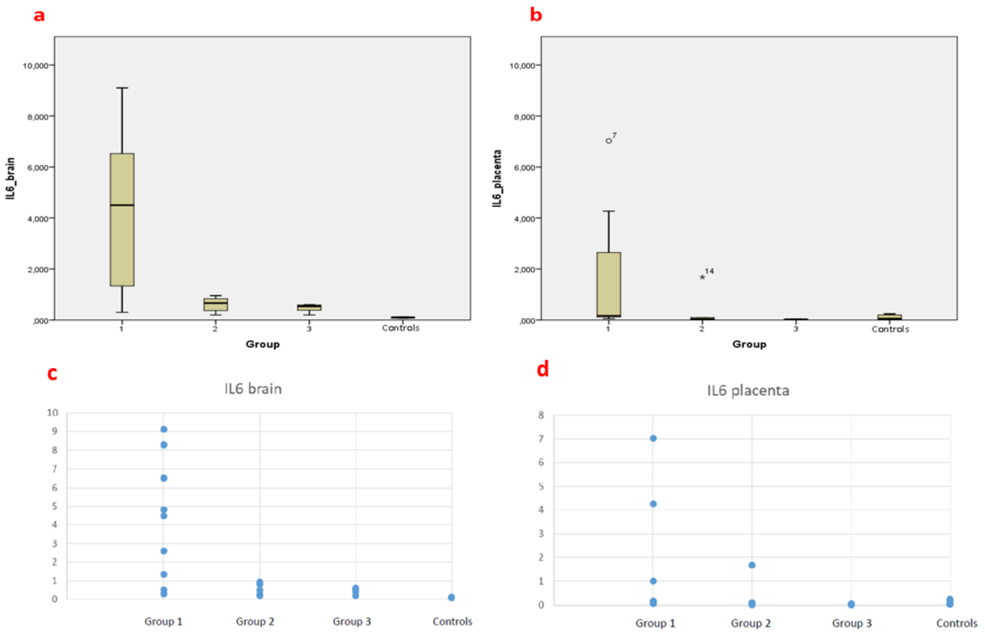

Figure S5: Box plots **(a-b)** and Dot plots **(c-d)** of the immunohistochemical expression of Interleukin-6 (IL-6) among groups: Brain cortex (a-c); Placental tissue (b-d). Data reported as median with interquartile range (IQR) of the percentage of the number of positive colored cells/microscopic area analyzed. P values are calculated by Kruskal-Wallis test. Comparisons between two independent groups were made by Mann-Whitney U test. In the brain cortex, IL-6 showed the highest expression in Group_1 B, while Group_2 B and Group_3B showed weak and similar immunoreactivity; in placenta tissue, the IL-6 expression was higher in Group_1 P than Group_2 P and Group_3 P.

S5a. Kruskal-Wallis test: $p<0.05$; Mann-Whitney U test: Group 1, Group 2, Group 3 vs controls: $p<0.05$. Group 1 vs Group 2: $p<0.05$; Group 1 vs Group 3: $p<0.05$

S5b. Kruskal-Wallis test: $p<0.05$; Mann-Whitney U test: Group 1 vs Group 3: $p<0.05$.
